# A new species of the spider genus *Selenocosmia* (Araneae, Theraphosidae) from Fujian, China

**DOI:** 10.3897/BDJ.10.e82406

**Published:** 2022-04-06

**Authors:** Ye-Jie Lin, Haifeng Chen, Xunyou Yan

**Affiliations:** 1 Hebei Key Laboratory of Animal Diversity, College of Life Science, Langfang, China Hebei Key Laboratory of Animal Diversity, College of Life Science Langfang China

## Abstract

**Background:**

The genus *Selenocosmia* Ausserer, 1871 includes 39 species. Five species were known from China. This genus has not been found in south-eastern China.

**New information:**

A new species of the genus *Selenocosmia* Ausserer, 1871 is described from China: *Selenocosmiazhangzhengi* Lin, **sp. n.** from Fujian. Photos and a morphological description of the new species are given. The type specimen of the new species is deposited in the Institute of Zoology, Chinese Academy of Sciences in Beiing (IZCAS).

## Introduction

The spider family Theraphosidae Thorell, 1869 includes 1031 species in 153 genera, with 39 species in the genus *Selenocosmia* Ausserer, 1871 (Li et al. 2021, Li 2020, Yao et al. 2021, Zhao et al. 2022, World Spider Catalog 2021). *Selenocosmia* can be distinguished from all other Selenocosmiinae genera by the presence of an apical keel reduced and shallow, basal lobe of retrolateral embolus keel reduced or absent; maxillary stridulatory lyra well-developed, lyra setae scimitar-shaped; tibia IV not incrassate and absence of dense penicillate retrolateral setal brushes on tibia and metatarsus IV (Yu et al. 2021).

Five *Selenocosmia* species were known from China, *S.anubis* Yu et al., 2021 (Yunnan); *S.jiafu* Zhu & Zhang, 2008 (Yunnan); *S.longiembola* Yu et al., 2021 (Yunnan); *S.qiani* Yu et al., 2021 (Guangdong) and *S.xinhuaensis* Zhu & Zhang, 2008 (Yunnan). Here, we report one new species: *S.zhangzhengi* sp. n. from Longyan, Fujian.

## Materials and methods

All specimens were preserved in 75% ethanol. Spermathecae were cleared in trypsin enzyme solution to dissolve non-chitinous tissues. Specimens were examined under a LEICA M205C stereomicroscope. Photomicroscope images were taken with an Olympus C7070 zoom digital camera (7.1 megapixels). Photographs were stacked with Helicon Focus 6.7.1 and processed in Adobe Photoshop CC 2018.

The terminology used in the text and figures follows Bertani (2000) and Sherwood et al. (2021). All measurements are in millimetres. Eye sizes were measured as the maximum diameter from either the dorsal or frontal view. Leg measurements are given as follows: total length (femur, patella, tibia, metatarsus, tarsus).

Abbreviations: **A** apical keel; **ALE** anterior lateral eyes; **AME** anterior median eyes; **BL** basal lobe of retrolateral embolus keel; **MOA** median ocular area; **PLE** posterior lateral eyes; **PME** posterior median eyes; **PS** prolateral superior keel; The type material is deposited in the Institute of Zoology, Chinese Academy of Sciences in Beijing (**IZCAS**).

## Taxon treatments

### Selenocosmia zhangzhengi

Lin sp. n.

1D3891FB-078A-5751-8A05-E700CB917571

BC40E565-96DC-4E66-9448-37AC9E6F67E5

#### Materials

- **Type status:** Holotype. **Occurrence:** recordedBy: Zheng Zhang; individualID: IZCAS-Ar42680; individualCount: 1; sex: male; **Taxon:** scientificName: *Selenocosmiazhangzhengi*; order: Araneae; family: Theraphosidae; **Location:** country: China; stateProvince: Fujian; verbatimLocality: Longyan, Xinluo District, Hengkengtou; verbatimElevation: 1143 m; verbatimLatitude: 25.3465°N; verbatimLongitude: 117.1205°E; **Event:** year: 2021; month: 6; day: 13
- **Type status:** Paratype. **Occurrence:** recordedBy: Chuan Liu, Linrui Yu, Xiaohan Ye, Zheng Zhang; individualID: IZCAS-Ar42681, Ar42682; individualCount: 2; sex: 2 females; **Taxon:** scientificName: *Selenocosmiazhangzhengi*; order: Araneae; family: Theraphosidae; **Location:** country: China; stateProvince: Fujian; locality: Longyan, Xinluo District, Hengkengtou; verbatimElevation: 1143 m; verbatimLatitude: 25.3465°N; verbatimLongitude: 117.1205°E; **Event:** year: 2021; month: 7; day: 11

#### Description

**Figure 1.**
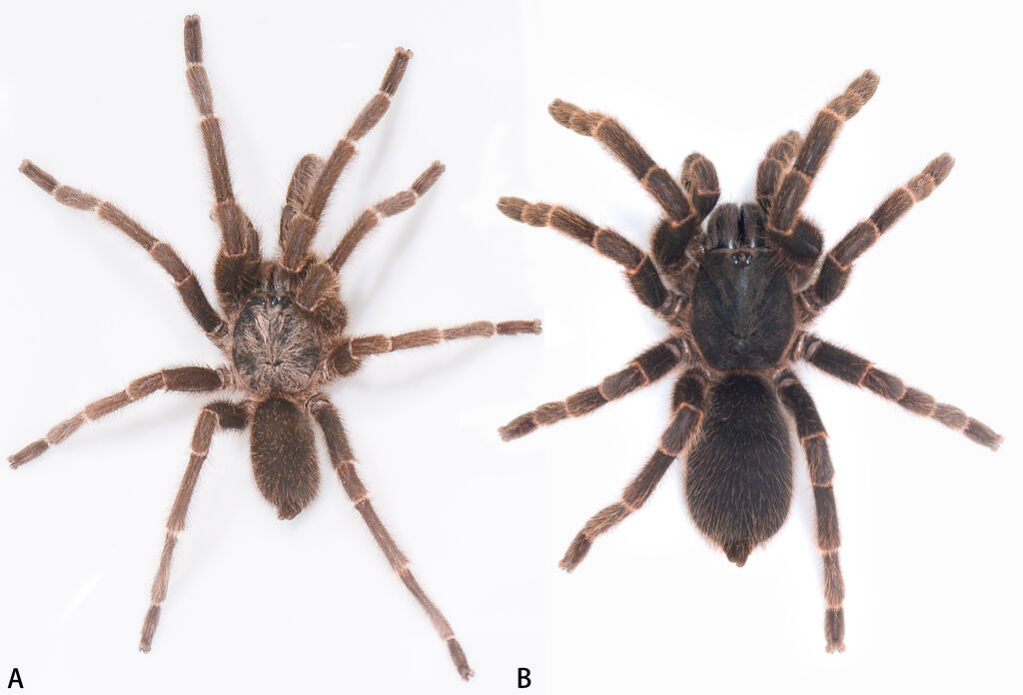
*Selenocosmiazhangzhengi* sp. n., live. **A** holotype male; **B** paratype female.

**Figure 2.**
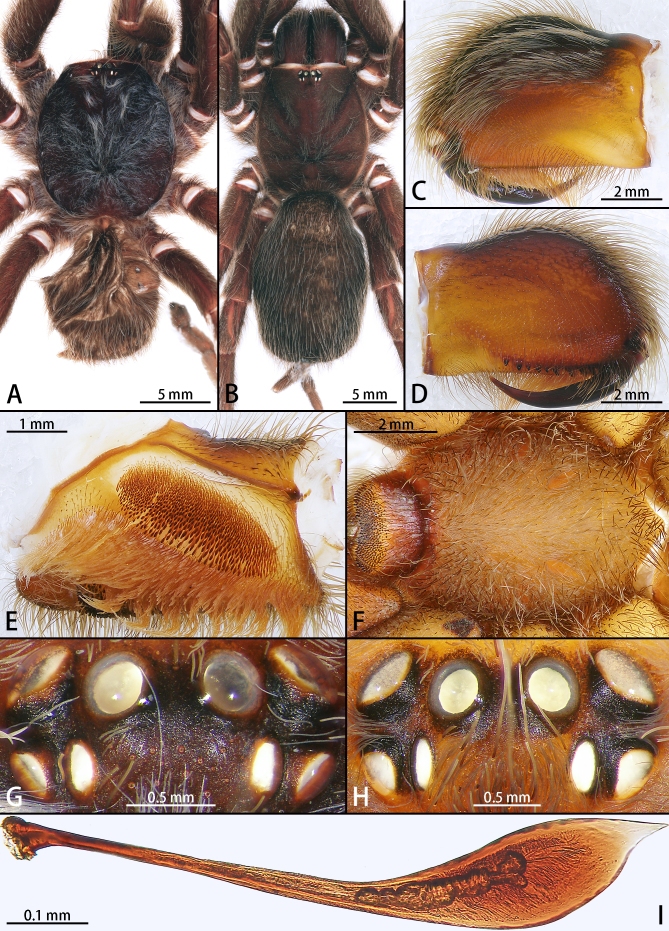
*Selenocosmiazhangzhengi* sp. n., holotype male (A, C–G) and paratype female (B, H, I). **A** male habitus; **B** female habitus; **C** chelicerae, retrolateral view; **D** prolateral view; **E** left palp maxillae; **F** sternum; **G, H** ocular tubercle; **I** stridulatory lyra.

**Male** (holotype, IZCAS-Ar42680) (Fig. 1A). Total length (without chelicerae) 22.65, carapace 12.12 long, 10.51 wide, dark brown with long setae. Opisthosoma brown, hirsute. Eye group 1.86 long, 0.95 wide. MOA 0.92 long, anterior width 1.14, posterior width 1.30. Eye sizes and interdistances: ALE 0.21, AME 0.39, PLE 0.15, PME 0.19; ALE–AME 0.18, AME–AME 0.27, PLE–PME 0.11, PME–PME 0.94. Fovea slightly procurved. Chelicerae dark brown, with row of 10 promarginal teeth, 32 mesoventral denticles. Labium wider than long, with ca. 237 cuspules. Sternum yellow brown with 3 pairs of sigilla (Fig. 2F). Legs with long and short setae. Tarsus I–III with 2 claws, tarsus IV with 3 claws, denticle number: (I 1, II 2, III 1–2, IV 1). Leg measurements: I 37.59 (12.32 + 4.88 + 7.65 + 7.21 + 5.53), II 32.29 (9.48 + 4.07 + 6.54 + 7.19 + 5.01), III 29.02 (8.51 + 4.72 + 4.81 + 6.51 + 4.47), IV 38.37 (11.12 + 4.75 + 7.82 + 9.08 + 5.60). Leg formula: 1423.

**Figure 3.**
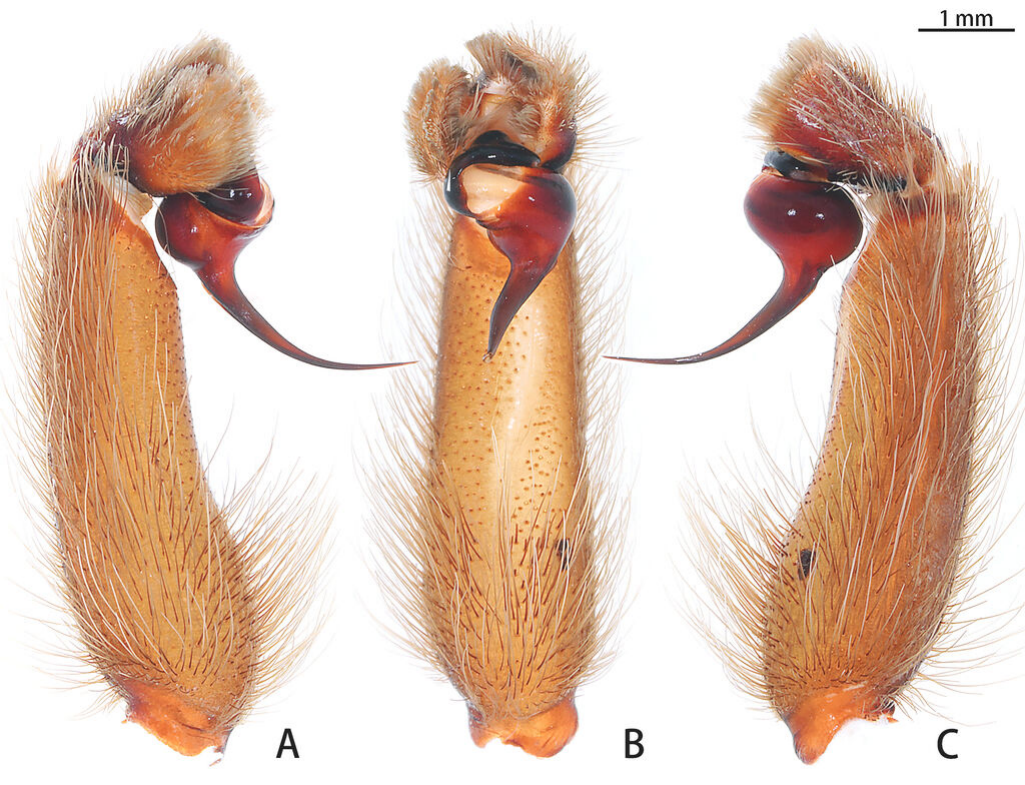
*Selenocosmiazhangzhengi* sp. n., holotype, male left palp **A** prolateral view; **B** ventral view; **C** retrolateral view.

Male palpal bulb (male palp with bulb (Fig. 3), male palpal bulb (Figs 4b, 5b, 6b, 7b, 8b, 9b). Maxillae with lyra setae, with ca. 372 cuspules ventrally. Tibia with many setae laterally, swollen at base. Bulb oval, embolus slightly curved, slender, sickle shaped, with A and PS. Distal edge of embolus relatively flat.

**Female** (paratype, IZCAS-Ar42638) (Fig. 1B). Total length (without chelicerae) 28.62, carapace 13.81 long, 10.08 wide, dark brown with setae. Fovea slightly procurved. Opisthosoma 16.41 long, 10.34 wide, oval, grey, hirsute. Eye group 2.25 long, 1.17 wide. MOA 1.11 long, anterior width 1.14, posterior width 1.30 (Fig. 2H). Eye sizes and interdistances: ALE 0.58, AME 0.41, PLE 0.40, PME 0.43; ALE–AME 0.17, AME–AME 0.28, PLE–PME 0.16, PME–PME 0.97. Fovea slightly procurved. Chelicerae with row of 12 promarginal teeth, 36 mesoventral denticles. Labium with ca. 299 cuspules. Palp maxillae with lyra setae, with ca. 468 cuspules ventrally. Tarsus I–III with 2 claws, tarsus IV with 3 claws, denticle number: (I 1, II 1, III 1, IV 1–3). Leg measurements: I 34.87 (9.52 + 4.78 + 10.53 + 5.32 + 4.72), II 29.04 (8.61 + 4.94 + 6.14 + 4.83 + 4.52), III 29.81 (8.02 + 4.46 + 8.47 + 4.54 + 4.32), IV 37.94 (9.63 + 5.61 + 10.21 + 7.41 + 5.08). Leg formula: 4213.

**Figure 10.**
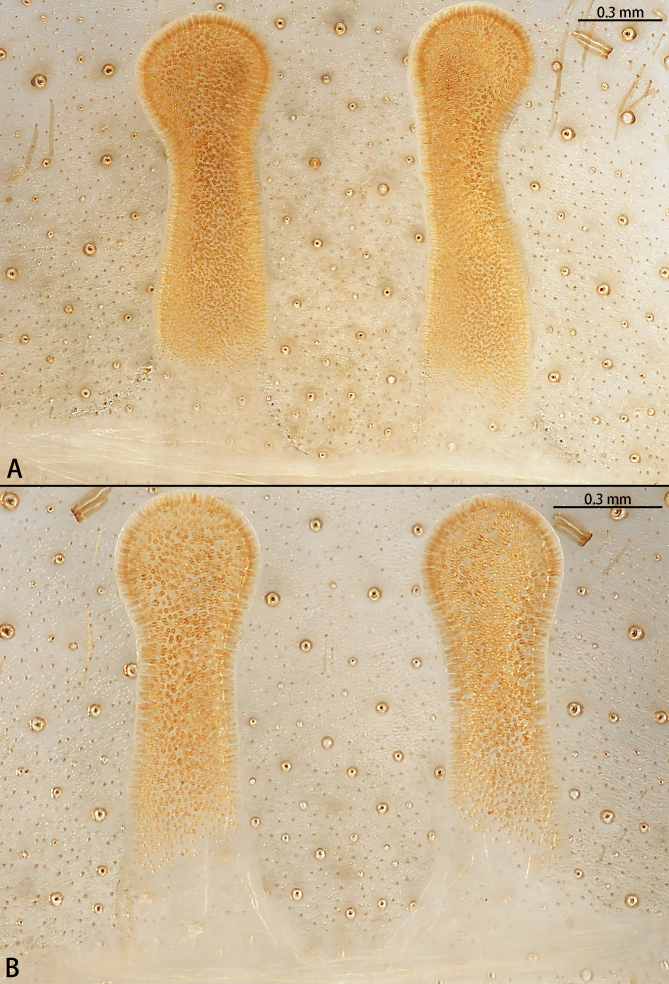
*Selenocosmiazhangzhengi* sp. n., vulva, dorsal view. Genitalia variation of paratypes.

Female genitalia (Fig. 10) simple. Spermathecae unilobed and long, swollen distally, without wrinkles.

##### Comparative material studied

*Selenocosmiajiafu* Zhu & Zhang, 2008: 2♂ (IZCAS), China: Yunnan: Xishuangbanna, Mengla County, Menglun Township，27.IX.2021, Conghao Yang leg.

##### Variation

Female (Paratypes; N2): body length: without chelicerae 20.14–28.62. Carapace 9.82–13.81 length, 7.16–10.08 wide. Chelicerae with row of 10–12 promarginal teeth, 34–36 mesoventral denticles. Labial cuspules 239–299.

#### Diagnosis

*Selenocosmiazhangzhengi* sp. n. is similar to males of *S.jiafu* Zhu & Zhang, 2008 and *S.qiani* Yu, S. Y. Zhang, F. Zhang, Li & Yang, 2021 by having the same angle of the embolus relative to the bulb. However, *S.zhangzhengi* sp. n. can be separated from *S.qiani* by the racket-shaped lyra setae on the maxillae (vs. dagger-shaped in *S.qiani*). Males of *S.zhangzhengi* sp. n. can be distinguished from *S.jiafu* by the absence of long, white setae on the tibia and metatarsus of the legs (vs. present in *S.jiafu*), the tip of the embolus is at an obtuse angle in *S.zhangzhengi* sp. n. (vs. acute angle in *S.qiani*) and small ventral lamina are absent on the distal embolus (Figs 8b, 9b) (vs. present in *S.jiafu* (Figs 8a, 9a). BL expanded, terminal blunt round in *S.zhangzhengi* sp. n. (Fig. 4b) (vs. terminal right angle in *S.jiafu* (Fig. 4a)). Females of *S . zhangzhengi* sp. n. can be differentiated from *S.jiafu* and *S.qiani* by the straight spermathecae (vs. curved in *S.qiani*) and the ratio of the length of the spermathecae to the distance between the spermathecae is almost 2–3:1 (Fig. 10) (vs. 3:2 in *S.jiafu* (Zhu and Zhang 2008)).

#### Etymology

The species is named after Mr. Zheng Zhang, who collected the type material; noun (name) in genitive case.

#### Distribution

Known only from the type locality.

#### Ecology

Retreats in burrows made in soil mixed with gravel, the burrows are usually about 3 to 4 cm in diameter. The spider web extends 10 to 20 cm inwards from the burrow. The spider moults inside. At night, the spider waits at the mouth of the burrow for its prey to pass by.

## Supplementary Material

XML Treatment for Selenocosmia zhangzhengi

## Figures and Tables

**Figure 4a.**
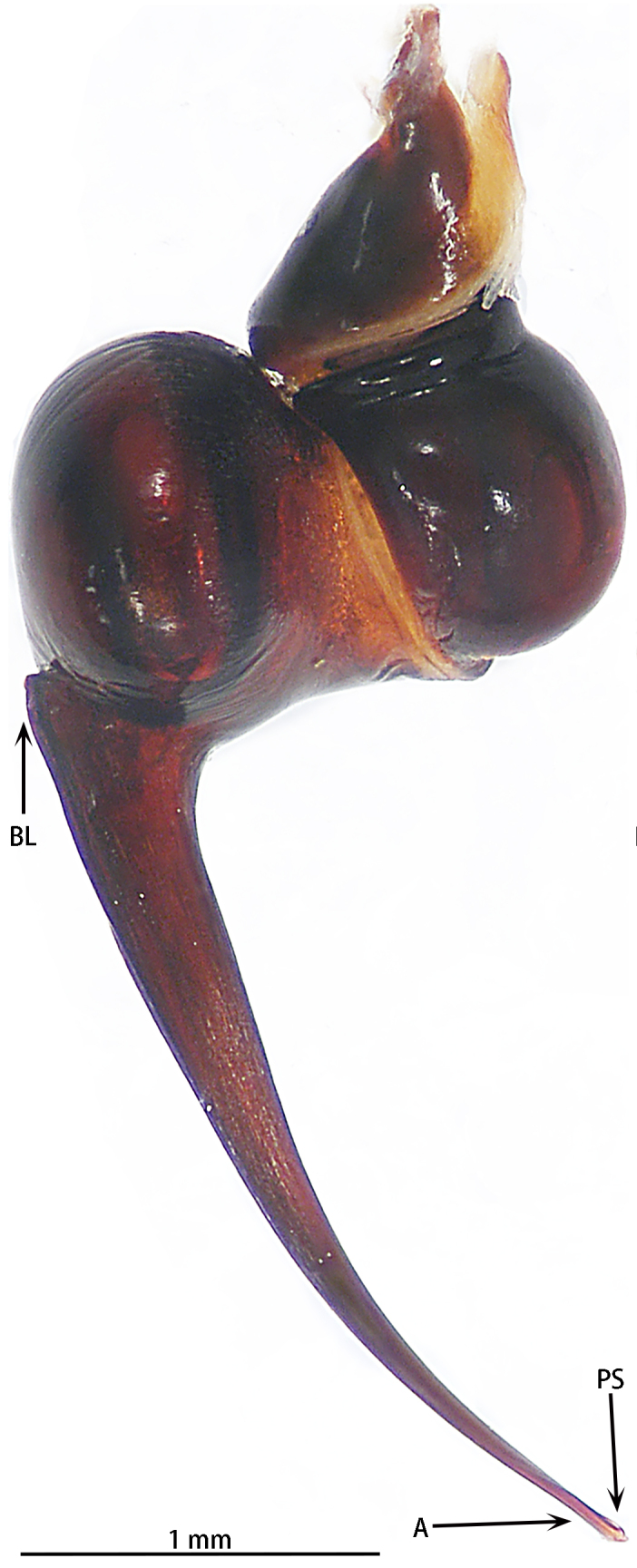
*Selenocosmiajiafu* Zhu & Zhang, 2008

**Figure 4b.**
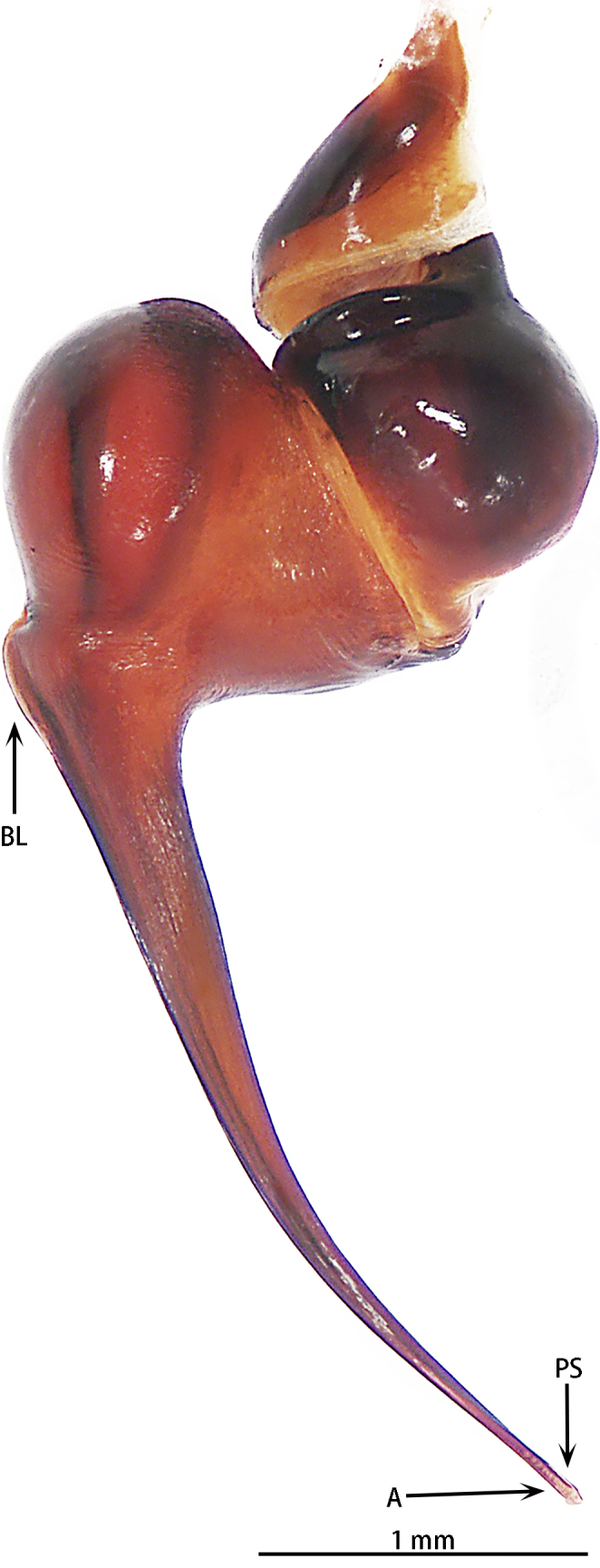
*Selenocosmiazhangzhengi* sp. n., holotype

**Figure 5a.**
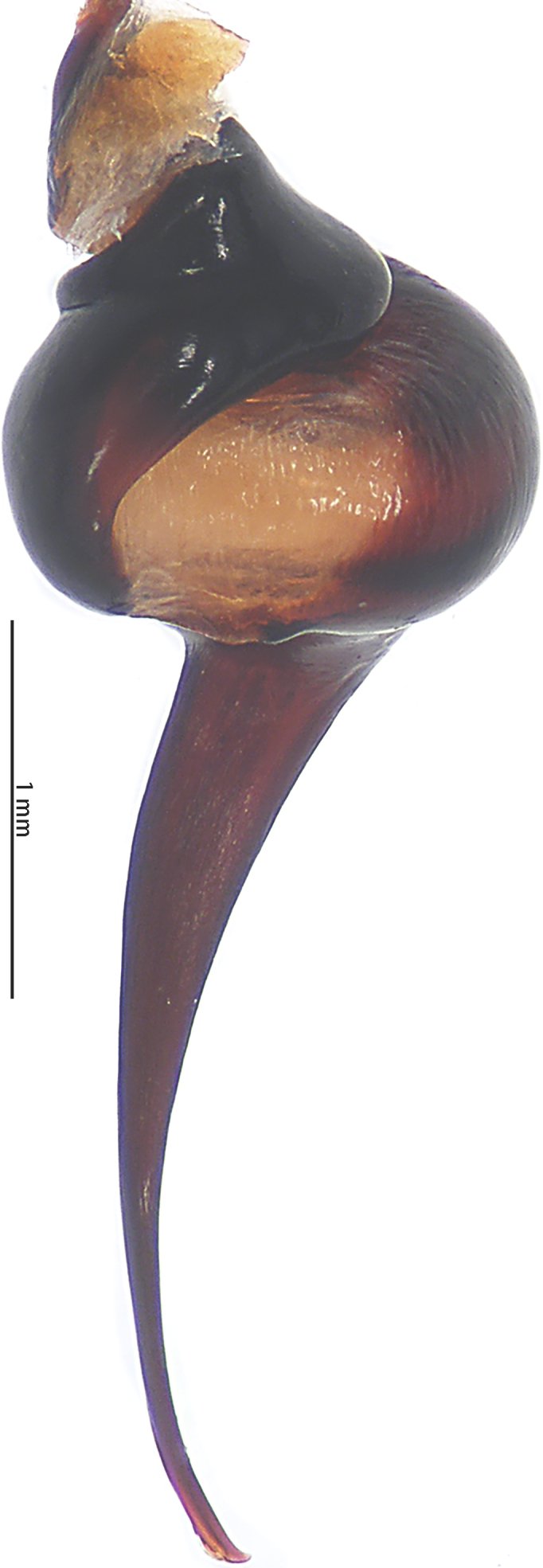
*Selenocosmiajiafu* Zhu & Zhang, 2008

**Figure 5b.**
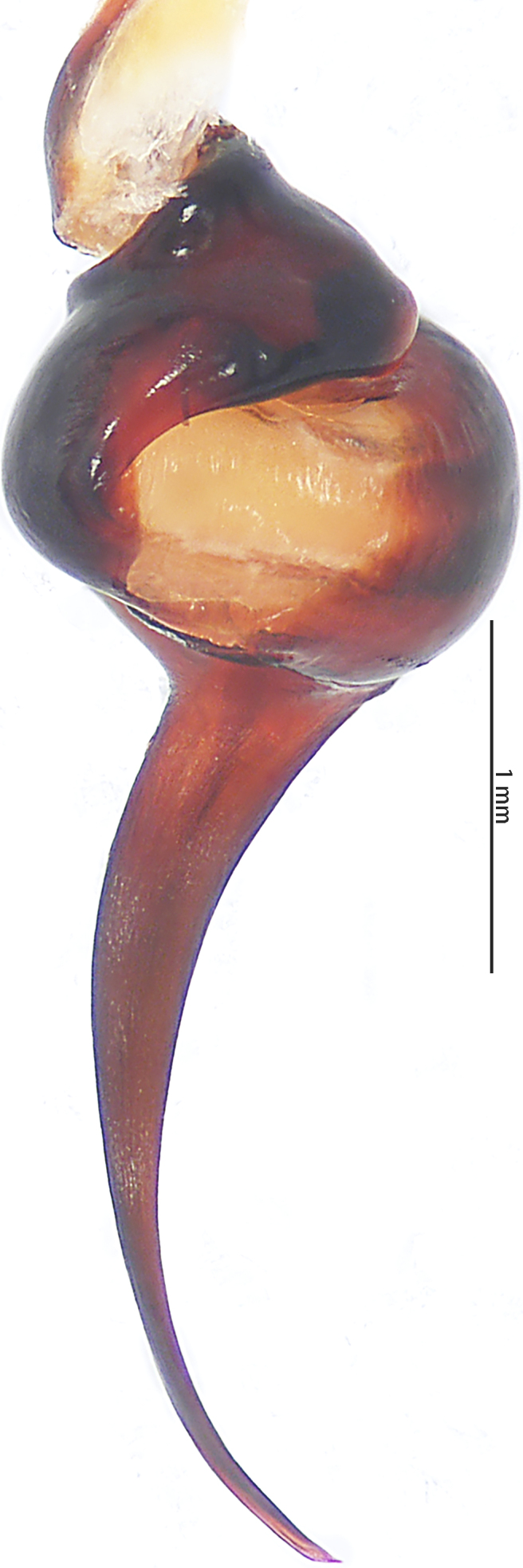
*Selenocosmiazhangzhengi* sp. nov., holotype

**Figure 6a.**
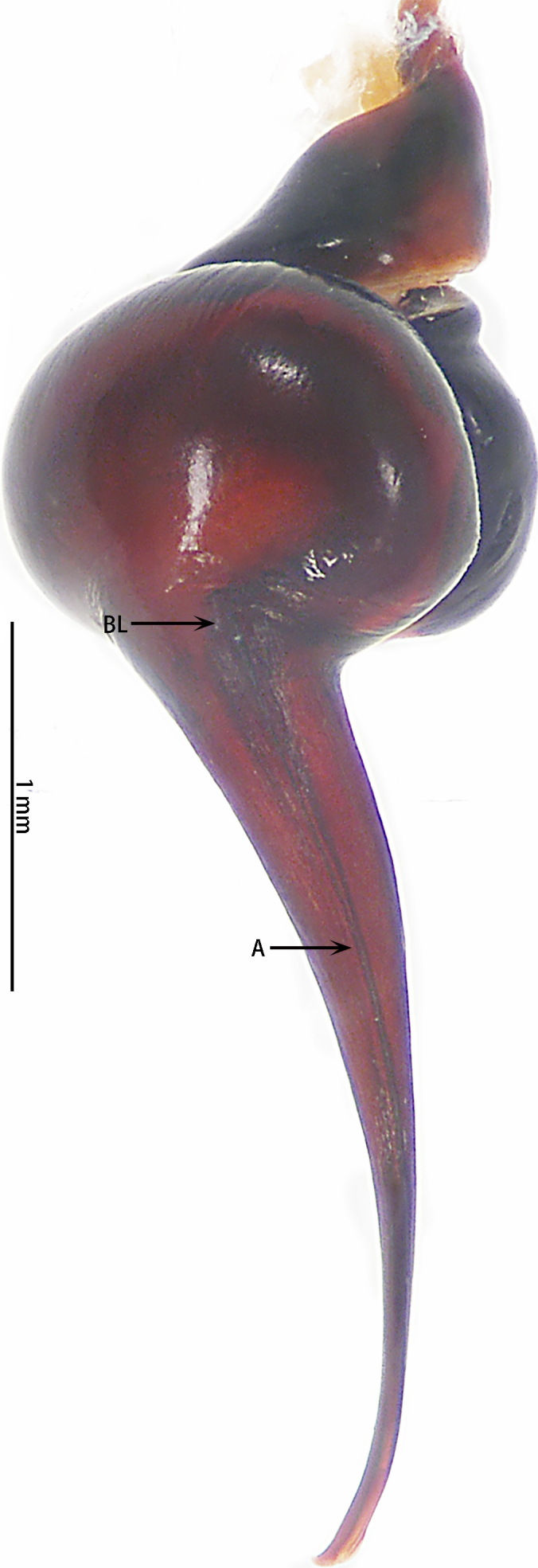
*Selenocosmiajiafu* Zhu & Zhang, 2008

**Figure 6b.**
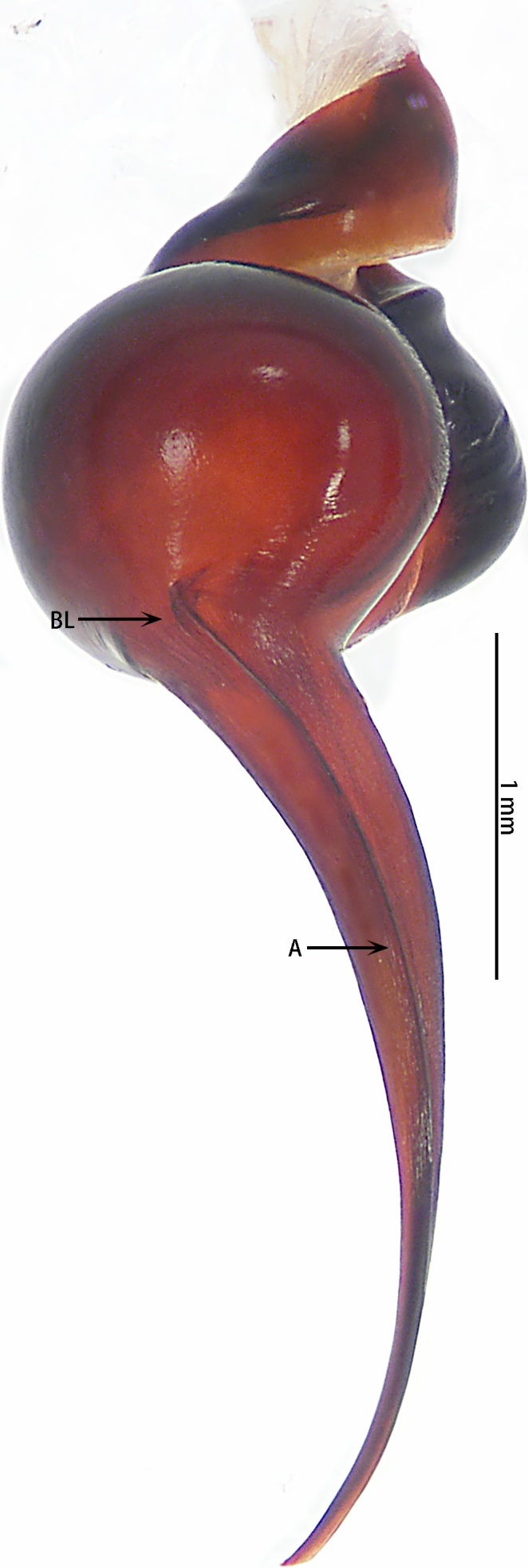
*Selenocosmiazhangzhengi* sp. n., holotype

**Figure 7a.**
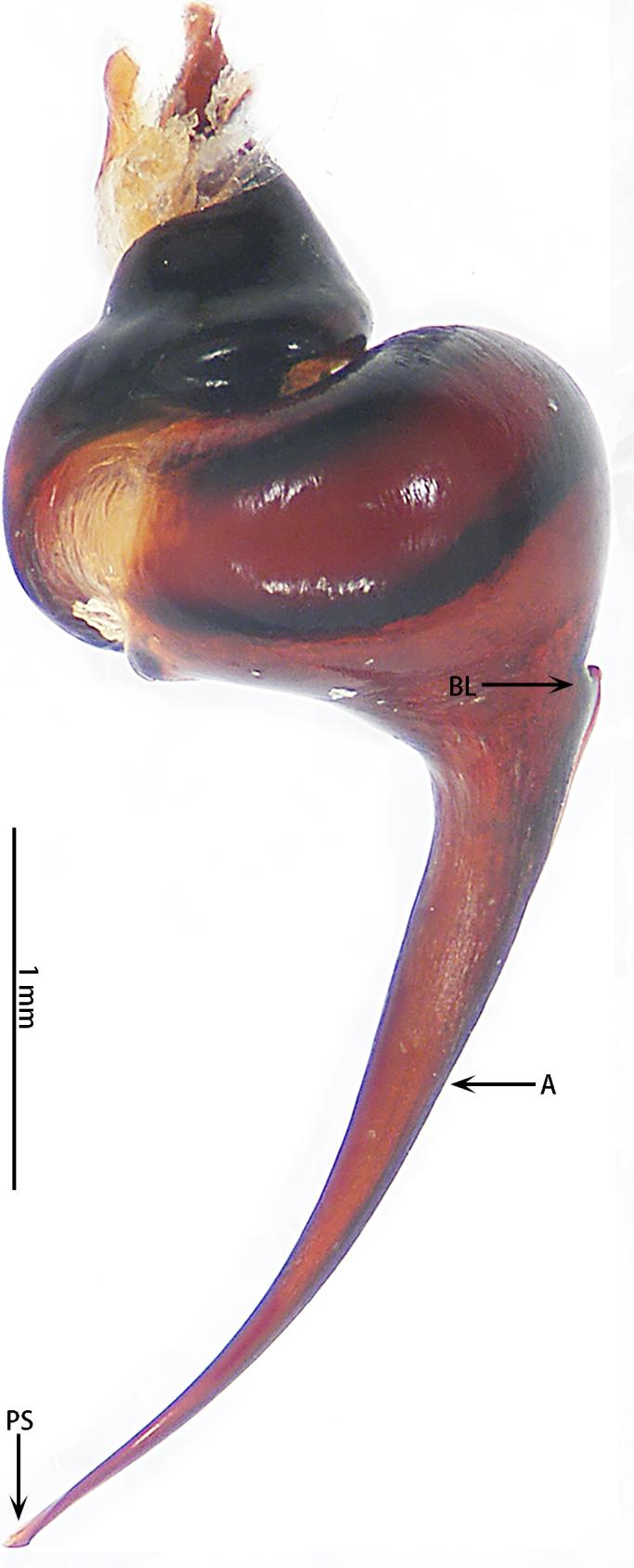
*Selenocosmiajiafu* Zhu & Zhang, 2008

**Figure 7b.**
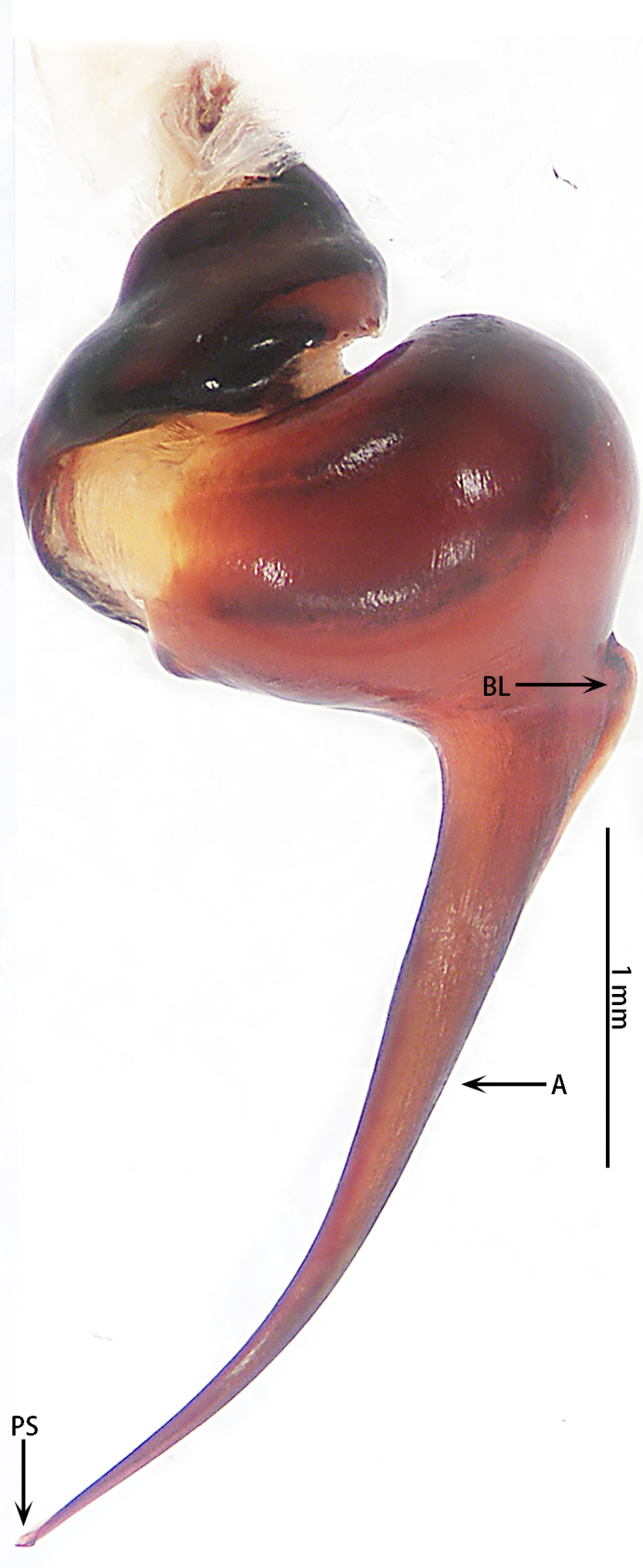
*Selenocosmiazhangzhengi* sp. n., holotype

**Figure 8a.**
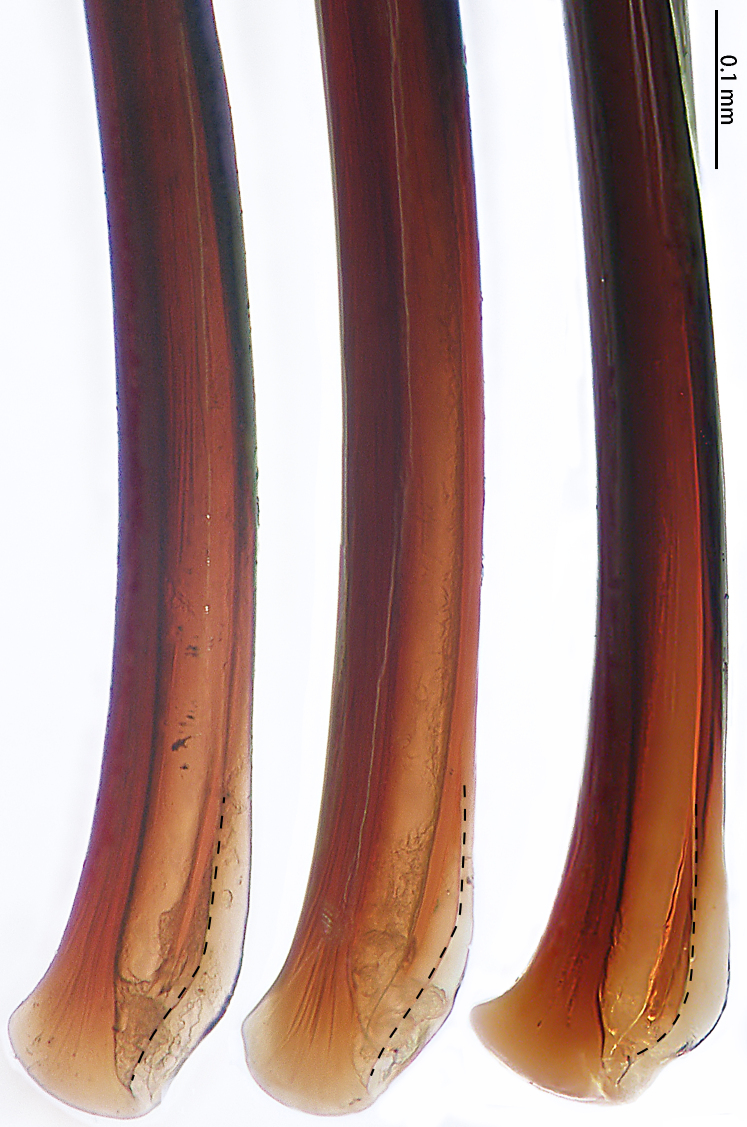
*Selenocosmiajiafu* Zhu & Zhang, 2008, individual variations

**Figure 8b.**
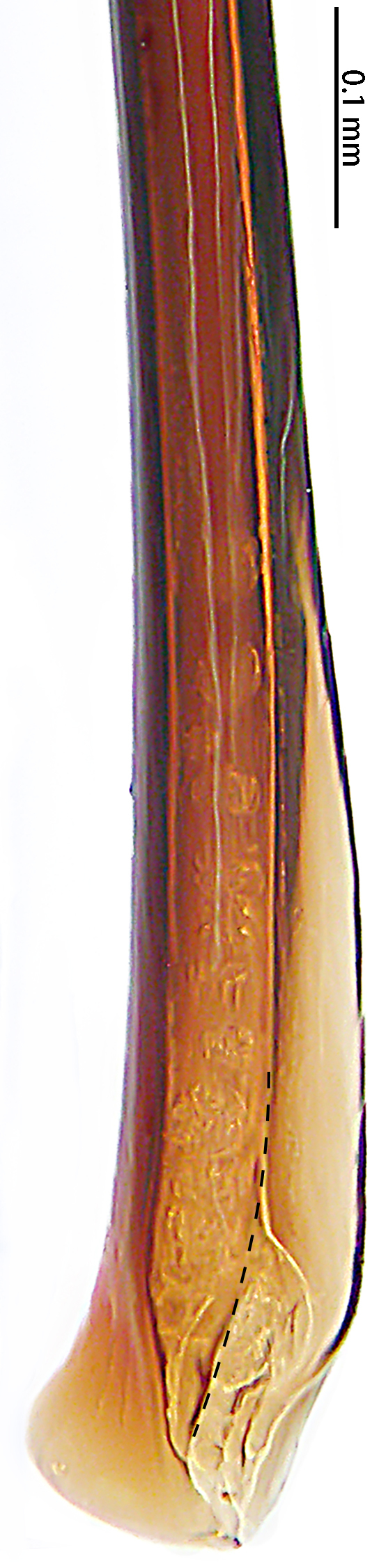
*Selenocosmiazhangzhengi* sp. n., holotype

**Figure 9a.**
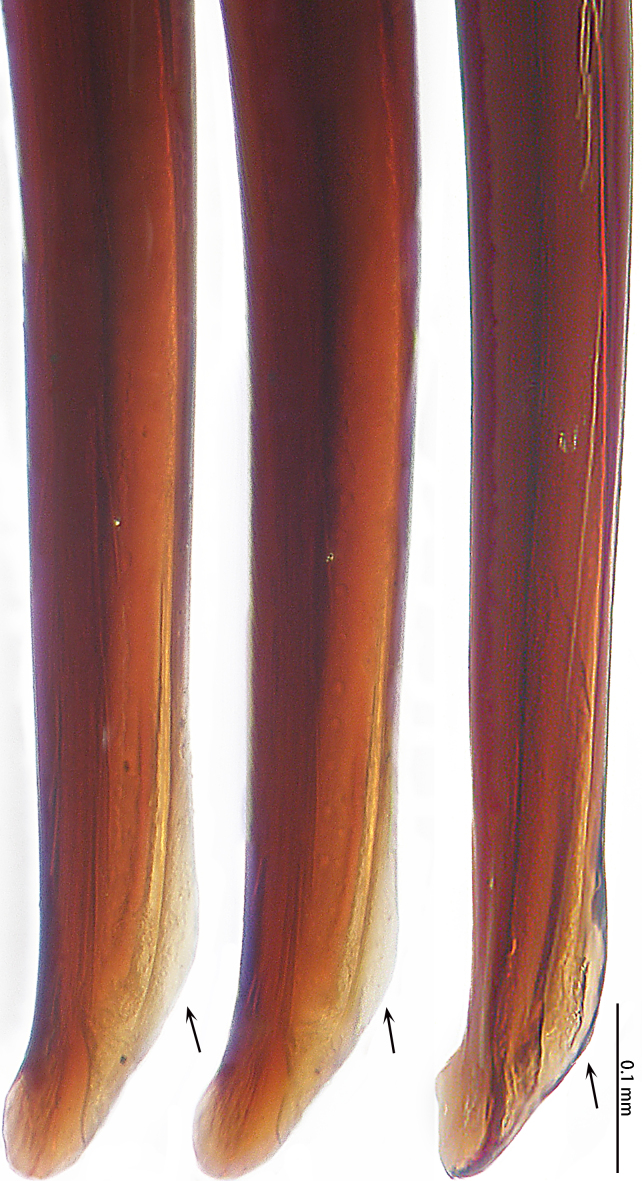
*Selenocosmiajiafu* Zhu & Zhang, 2008, individual variations

**Figure 9b.**
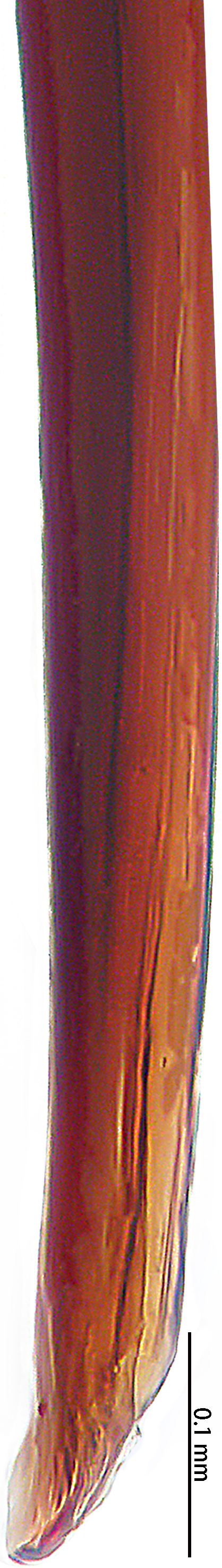
*Selenocosmiazhangzhengi* sp. n., holotype
